# Gender Gap in Scientific Publications on COVID-19 in Italy During the First Wave of the Pandemic: An Observational Study

**DOI:** 10.3389/fpubh.2022.818594

**Published:** 2022-06-30

**Authors:** Elena Mazzalai, Federica Turatto, Corrado De Vito

**Affiliations:** Department of Public Health and Infectious Diseases, Sapienza University of Rome, Rome, Italy

**Keywords:** science of science, gender gap, academia, COVID-19, Italy

## Abstract

**Background:**

Worldwide, concerns rise on how COVID-19 pandemic impacted heavily on women, even on those belonging to the scientific community. The Italian scientific production regarding the COVID-19 throughout the first months of the health emergency could help to understand the heft of female researchers in this unique period.

**Objectives:**

This study aims to investigate the gender gap in the scientific production on COVID-19 in Italy during the first months of the pandemic.

**Methods:**

A systematic search of the literature was conducted and, for each included study, first and last author's gender, type of study, number of co-authors, type of affiliation, journal's Impact Factor (IF) and specialization were extracted. Descriptive and univariate analyses were performed.

**Results:**

22.2% of the articles were signed by a woman as first author, 18.1% as last authors. Female authorship was less frequent than male authorship regardless of the type of study, number of co-authors, type of affiliation and field of specialization.

**Conclusion:**

This analysis reveal a low prevalence of studies with a female first or last author and suggests that the low share of female authors publishing on COVID-19 during the considered timespan is a transversal issue throughout the Italian medical field.

## Introduction

The COVID-19 pandemic had a high impact on Italy, especially in the first months of 2020. A national lockdown was implemented from March 9th and lasted more than 2 months (1). During this period the country had been severely hit and the Italian National Healthcare Service suffered an overwhelming pressure. The national scientific community reacted promptly to the emergency: at the beginning of the pandemic Italy was one of the countries publishing the most on this topic (2).

However, global scientific literature is unanimous in bringing out how the publication rate during the health emergency was not consistent when looking at authors' gender: the share of women as first author declined compared to the same period of 2019 (3, 4). The issue of low female authorship in scientific publication is anything but a new discovery, both in Italy (5) and worldwide (6), where female authors only count for 30% of all signatures in the scientific field. Even though the gender gap in publication has been shrinking, acknowledged factors hindering women academic productivity still persist (7, 8). Some of these, such as the higher burden of domestic duties and children care, became even more visible and of public interest after the outbreak of COVID-19 pandemic and the implementation of the preventive measures (9–11).

Based on these assumptions, this article aims at investigating the gender gap in the scientific production on COVID-19 in Italy in the first months of the pandemic using data from a previous research (12).

## Methods

The analysis was performed on the scientific literature regarding the COVID-19 outbreak in Italy. A systematic search of the literature in accordance with the Preferred Reporting Items for Systematic Reviews and Meta-Analyses (PRISMA) 2009 Statement was performed (13). A comprehensive search strategy was developed to identify articles published from December 2019 to 24th of April 2020 which included the terms (“COVID” OR “SARS-CoV-2” OR “coronavirus”) AND (“Italy” OR “Italian”) in their title and/or abstract. Papers published by first authors with an Italian affiliation were included, with no restriction based on language or study design. In particular, for each included study, first author's gender, last author's gender, type of study, number of co-authors, type of affiliation, journal's Impact Factor (IF) and specialization were extracted. Gender was classified as “female” and “male” and was assigned based on the name of the author. For names which can be used for both males and females, gender was assigned according to the most common use of the name. When the author was a scientific society or an institution, gender was classified as “not attributable.” Specialization was assigned based on the first author's affiliation, and was further categorized into four fields: Surgical, Medical, Public health, Non-medical. Legal medicine was included in the Medical field. Statistical specializations were included in the Public Health field. The Non-medical field included studies of anthropology, psychology, and ethics. Type of affiliation was categorized as either academic or non-academic based on first author's affiliation. The impact factor for each journal was obtained from the Journal of Citations Report 2019 (14).

Articles were classified according to study type based on an adaptation of the classification of studies in medical research developed by Röhrig et al. (12, 15).

Descriptive analyses were performed on Microsoft Excel, calculating the frequency of female first author and female last author for the included articles, median and interquartile range for continuous variables. Univariable and multivariable analyses were carried out on Stata 17 to investigate association between first and last author's gender and number of co-authors, type of study, journal IF, specialization and type of affiliation. Univariable analysis was performed using Chi-squared test and Fisher's Exact Test for categorical variables and Student's *t*-test or Mann Whitney's *U*-test for continuous variables, and logistic regression was used for the multivariable analysis. A threshold of *p* ≤ 0.05 was used to determine statistical significance.

## Results

A total of 205 articles were retrieved from the research. Two papers were signed by scientific societies. The gender of the first author was feminine in 45 papers (22.2%) and masculine in 158 (77.8%). Among the 188 articles written by more than one author, the last author's gender was feminine in 34 papers (18.1%) and masculine in 154 (81.9%). Eleven articles were signed by female researchers both as first and last author. This number is equal to a share of 5.9% of the 188 articles, of 32.4% of the studies with a woman as last author and 25.6% of those with a woman as first author.

Table 1 illustrates the distribution of articles by author's gender and specialization, type of study and affiliation. Regarding the specialization field extracted from the first authors' affiliation, the majority of the papers were classified in the Medical. Among these papers, 23.0% had a female first authors and 21.3% had a female last author. Whether the specialization field was Medical, Surgical, Non-medical, or related to Public health, no significance difference was found for gender distribution of neither first nor last author in the univariable analysis. However, when controlling for all other variables, articles with surgical specialization had significantly lower odds of female authorship compared to articles with public health specialization (OR = 0.16, *p* = 0.03).

**Table 1:** Number and percentage of articles published according to specialization, type of study, and affiliation by author's gender.

		**First authorship**				**Last authorship**		
		**Female**	**Male**			**Female**	**Male**	
	***N***	**% (*n*)**	**% (*n*)**	***P***	***N***	**% (*n*)**	**% (*n*)**	***P***
**Specialization**
Surgical	35	14.3 (5)	85.7 (30)	0.51^a^	34	5.9 (2)	94.1 (32)	0.17^a^
Medical	135	23.0 (31)	77.0 (104)		127	21.3 (27)	78.7 (100)	
Public health	16	31.3 (5)	68.7 (11)		15	20.0 (3)	80.0 (12)	
Non-medical	15	26.7 (4)	73.3 (11)		10	20.0 (2)	80.0 (8)	
Total^b^	201	22.4 (45)	76.6 (156)		186	18.3 (34)	81.7 (152)	
**Type of study**
Primary	137	23.4 (32)	76.6 (105)	0.55	132	15.9 (21)	84.1 (111)	0.24
Secondary	11	9.1 (1)	90.9 (10)		10	10.0 (1)	90.0 (9)	
Other	55	21.8 (12)	78.2 (43)		46	26.1 (12)	73.9 (34)	
Total^b^	203	22.2 (45)	77.8 (158)		188	18.1 (34)	81.9 (154)	
**Type of primary study**
Basic	5	60.0 (3)	40.0 (2)	0.06^a^	5	40.0 (2)	60.0 (3)	0.36^a^
Clinical	27	33.3 (9)	66.7 (18)		26	19.2 (5)	80.8 (21)	
Epidemiological	19	26.3 (5)	73.7 (14)		18	11.1 (2)	88.9 (16)	
Management	86	17.4 (15)	82.6 (71)		83	14.5 (12)	85.5 (71)	
Total^b^	137	23.4 (32)	76.6 (105)		132	15.9 (21)	84.1 (111)	
**Affiliation**
Academic	102	20.6 (21)	79.4 (81)	0.59	94	14.9 (14)	85.1 (80)	0.26
Non academic	101	23.8 (24)	76.2 (77)		94	21.3 (20)	78.7 (74)	
Total^b^	203	22.2 (45)	77.8 (158)		188	18.1 (34)	81.9 (154)	

a *Fisher's exact p*.

b *The analyses consider papers provided with the listed variables and information on author's gender. Papers signed by a single author are not considered in the analyses on last authorship*.

The distribution of male and female first and last authorship does not seem to be influenced in a statistical significance way by type of study (*p* = 0.55 and *p* = 0.24, respectively), nor by the type of primary study, which count for the most published type of study (*p* = 0.06 and *p* = 0.36, respectively).

Academic and non-academic affiliation is equally distributed, with 102 first authors presenting an academic affiliation and 101 a non-academic one. Last authorship is equally divided between academic (94) and non-academic (94). Once again, the gender distribution of first and last authorship do not vary according to the affiliation type, presenting no statistical significant differences (*p* = 0.59 and *p* = 0.26 for first and last author's gender, respectively).

Table 2 shows the IF of the journal and number of co-authors of articles published according to author's gender. The number of co-authors does not vary significantly depending on the gender of neither first nor last author, with a median number of co-authors of 6 [IQR 3–11] for female and 5 [IQR 2–8] for male first authors (*p* = 0.16), and 4.5 [IQR 3–8] and 5 [IQR 2–10] for female and male last authors, respectively (*p* = 0.83). Therefore, the share of female authorship does not appear to influenced by the number of co-authors.

**Table 2:** Journal IF and number of co-authors by author's gender.

		**First authorship**				**Last authorship**		
		**Female**	**Male**			**Female**	**Male**	
	***N***	**Median [IQR]**	**Median [IQR]**	***P***	**N**	**Median [IQR]**	**Median [IQR]**	***P***
Journal IF	174	4.9 [2.8–7.3]	3.6 [2.3–6.5]	0.13	161	5.8 [3.6–7.1]	3.6 [2.4–6.8]	0.03
No. of co-authors	188	6 [3–11]	5 [2–8]	0.16	188	4.5 [3–8]	5 [2–10]	0.83

Looking at last authorship's gender, journals hosting female last signatures present on average statistically higher IF (Female last author: IF 5.8 [IQR 3.6–7.1]; male last author: IF 3.6 [IQR 2.4–6.8]; *p* = 0.03). Analogous findings did not appear when considering first authorship.

None of these factors appear to be significantly associated with first or last female authorship in the multivariable analysis.

## Discussion

This preliminary analysis of the scientific literature on COVID-19 in Italy during the first wave of the pandemic highlights a low prevalence of studies with a female first or last author across disciplines and institutions in the medical area during the first wave of the pandemic. Even though our results show a significant difference between articles published in the public health field and the surgical field, the share of female authors remained below 31.6% in all fields of specialization. Our analyses also show that the median journal IF was higher for articles with a female last author compared to those with a male last author and the difference was statistically significant in the univariable analysis, however the IF does not appear to be a key factor in the multivariable analysis.

A possible explanation for our results could be found in the low representation of females in the scientific and medical field in Italy. However, OECD data (16) show that women accounted for 43.4% of the physicians in Italy in 2019, with the highest share of 64.0% among physicians aged 35–44 years. Furthermore, an analysis of the composition of students in Italian universities in 2010 showed a higher prevalence of female students in areas such as Medicine and Pharmacy, although still representing only 30% of the students of scientific faculties (17). These data suggest that women are not a minority in the biomedical field, and do not seem to justify the lower authorship share which emerged from our analysis.

However, when considering academic career, data from Italian universities for the scientific fields related to “medical sciences” and “biological sciences” are not as encouraging: women account for 32.7% of the academic personnel in the “medical science” field. The share of women is inversely proportional to career advancement: women represent 46.2% of researchers, 33.5% of associate professors and only 19.1% of ordinary professors. In the field of “biological sciences,” women account for 54.2% of the total academic staff, but are overrepresented among researchers (62.2%), less represented but still the majority among associate professors (55.9%), and only 35.9% among ordinary professors (18). Our findings are therefore all the more alarming, since a low representation of female first and last author could indicate that women are missing out on the opportunity of career advancement during COVID-19 (19).

The low proportion of female-authored scientific literature on COVID-19 could have implications for society as a whole, as it could affect the overall quality and comprehensiveness of evidence, since women authors contribute to inclusion of gender-relevant and gender-specific issues (19).

While indicating that women in Italy have published less than their male colleagues during the COVID-19 pandemic, our data do not allow to draw conclusions on the impact of the pandemic on female academic productivity. However, other studies have shown that women's academic productivity has decreased during the pandemic in medical and non-medical fields (3, 4, 9, 20, 21). This was found to be true especially for COVID-related work (22, 23). Reasons for this have been identified in the share of home and family care responsibilities falling disproportionately on women during lockdown (4, 21, 24) and the increased workload related to teaching responsibilities for women academics (9, 25). The disproportionate impact on early research career women has been further associated with lower risk-taking attitude in taking on new research projects during the emergency phase (19, 26).

Further research on the topic is needed to gain a deeper understanding of the impact of the pandemic on publication trends in Italy.

Our study has some limitations. First of all, our study takes into consideration only published articles and does not account for submitted articles. It is therefore not possible to determine whether the lower share of female authorship is due to a lower number of submissions by female authors or to other steps of the publication process. Not accounting for submissions in such a limited time frame could also have led to leave out from our analysis papers submitted but not yet published at that time. However, this applies to all submitted papers, and is unlikely to influence the gender distribution in authorship. Furthermore, it has been pointed out that during the first months of pandemic, journals have accelerated the publication process on COVID-19 related papers, therefore reducing the time lag between submission and publication (27). The strategy used to assign gender for names could have led to misclassification of some authors' gender. Moreover, the specialization attribution could have led to misclassification on two accounts: the field of the affiliation could not correspond to the actual specialization of the first author; the specialization of the first author could not be the same as the last author's. Furthermore, our study is limited to a section of the scientific literature published on a specific topic in a limited time frame. The terms included in the search strategy could also have determined the exclusion of articles related to COVID-19 published by Italian authors which did not include “Italy” in the title or abstract. This could have particularly affected the inclusion of basic science articles.

We believe that highlighting the gender gap in publications related to COVID-19 in Italy is important for two reasons: first of all, it provides the opportunity to adapt the global discourse on gender inequalities during COVID-19 to a local context. Furthermore, the COVID-19 pandemic will have long-lasting consequences and bring about innovation in all domains of our lives. Hopefully, highlighting critical issues such as the one presented in this paper, could be a stimulus toward advancing gender equity in academia.

## Data Availability Statement

The raw data supporting the conclusions of this article will be made available by the authors, without undue reservation.

## Author Contributions

All authors conceived and designed the study and approved the final version of the manuscript. EM and FT collected and analyzed the data and wrote the initial draft. CD revised the manuscript.

## Conflict of Interest

The authors declare that the research was conducted in the absence of any commercial or financial relationships that could be construed as a potential conflict of interest.

## Publisher's Note

All claims expressed in this article are solely those of the authors and do not necessarily represent those of their affiliated organizations, or those of the publisher, the editors and the reviewers. Any product that may be evaluated in this article, or claim that may be made by its manufacturer, is not guaranteed or endorsed by the publisher.

## References

[1] del Consiglio dei Ministri,. Decreto del Presidente del Consiglio dei Ministri (2020). Presidenza del Consiglio dei Ministri. Available online at: https://www.gazzettaufficiale.it/eli/id/2020/03/09/20A01558/sg

[2] Odone A Salvati S Bellini L Bucci D Capraro M Gaetti G. The runaway science: a bibliometric analysis of the COVID-19 scientific literature: how COVID-19 has changed academic publishing. Acta Biomed. (2020) 91:34–9. 10.23750/abm.v91i9-S.10121 32701915 PMC8023084

[3] Andersen JP Nielsen MW Simone NL Lewiss RE Jagsi R. COVID-19 medical papers have fewer women first authors than expected. eLife. (2020) 9:e58807. 10.7554/eLife.58807 32538780 PMC7304994

[4] Vincent-Lamarre P Sugimoto CR Larivière V. The Decline of Women's Research Production During the Coronavirus Pandemic [Internet]. Natureintex.com (2020). Available online at: https://www.natureindex.com/news-blog/decline-women-scientist-research-publishing-production-coronavirus-pandemic (accessed June 23, 2021).

[5] Abramo G D'Angelo CA Caprasecca A. Gender differences in research productivity: a bibliometric analysis of the Italian academic system. Scientometrics. (2009) 79:517–39. 10.1007/s11192-007-2046-8

[6] Lavrière V Ni C Gingras Y Cronin B Sugimoto CR. Bibliometrics: global gender disparities in science. Nature. (2013) 504:2011–3. 10.1038/504211a 24350369

[7] Carnes M Morrissey C Geller SE. Women's health and women's leadership in academic medicine: hitting the same glass ceiling? J Women's Health. (2008) 17:1453–62. 10.1089/jwh.2007.0688 18954235 PMC2586600

[8] Rochon PA Davidoff F Levinson W. Women in academic medicine leadership: has anything changed in 25 years? Acad Med. (2016) 91:1053–6. 10.1097/ACM.0000000000001281 27306972

[9] Viglione G. Are women publishing less during the pandemic? Here's what the data say. Nature. (2020) 581:365–6. 10.1038/d41586-020-01294-9 32433639

[10] Roig R Aybar C Pavía JM. COVID-19, gender housework division and municipality size in Spain. Soc Sci. (2022) 11:37. 10.3390/socsci11020037

[11] Del Boca D Oggero N Profeta P Rossi M. Women's and men's work, housework and childcare, before and during COVID-19. Rev Econ Household. (2020) 18:1001–17. 10.1007/s11150-020-09502-1 32922242 PMC7474798

[12] Turatto F Mazzalai E Pagano F Migliara G Villari P De Vito C. A systematic review and bibliometric analysis of the scientific literature on the early phase of COVID-19 in Italy. Front Public Health. (2021) 9:666669. 10.3389/fpubh.2021.666669 34239853 PMC8258167

[13] Moher D Liberati A Tetzlaff J Altman DG. Preferred reporting items for systematic reviews and meta-analyses: the PRISMA statement. PLoS Med. (2009) 6:e1000097. 10.1371/journal.pmed.1000097 19621072 PMC2707599

[14] InCites Journal Citations Reports [Internet]. (2019). Available online at: https://jcr.clarivate.com/ (December 11, 2020).

[15] Röhrig B Prel JB du Wachtlin D Blettner M. Types of study in medical research: part 3 of a series on evaluation of scientific publications. Dtsch Aerztebl Online. (2009) 106:262–8. 10.3238/arztebl.2009.0262 19547627 PMC2689572

[16] OECD, Statistics [Internet],. Available online at: https://stats.oecd.org/ (accessed June 23, 2021).

[17] Noè C,. Genere e Scelte Formative. AlmaLaurea Working Papers [Internet] (2012). Available online at: http://www2.almalaurea.it/universita/pubblicazioni/wp/pdf/wp54.pdf

[18] Cerca, Università [Internet],. Available online at: https://cercauniversita.cineca.it/ (accessed June 23, 2021).

[19] Shamseer L Bourgeault I Grunfeld E Moore A Peer N Straus SE. Will COVID-19 result in a giant step backwards for women in academic science? J Clin Epidemiol. (2021) 134:160–6. 10.1016/j.jclinepi.2021.03.004 33705957 PMC9758692

[20] Cevik M Haque SA Manne-Goehler J Kuppalli K Sax PE Majumder MS. Gender disparities in coronavirus disease 2019 clinical trial leadership. Clin Microbiol Infect. (2021) 27:1007–10. 10.1016/j.cmi.2020.12.025 33418021 PMC7785275

[21] Krukowski RA Jagsi R Cardel MI. Academic productivity differences by gender and child age in science, technology, engineering, mathematics, and medicine faculty during the COVID-19 pandemic. J Women's Health. (2021) 30:341–7. 10.1089/jwh.2020.8710 33216682 PMC7957370

[22] Muric G Lerman K Ferrara E. Gender disparity in the authorship of biomedical research publications during the COVID-19 pandemic: retrospective observational study. J Med Intern Res. (2021) 23:e25379. 10.2196/25379 33735097 PMC8043146

[23] Pinho-Gomes AC Peters S Thompson K Hockham C Ripullone K Woodward M. Where are the women? Gender inequalities in COVID-19 research authorship. BMJ Glob Health. (2020) 5:e002922. 10.1136/bmjgh-2020-002922 32527733 PMC7298677

[24] Yildirim TM Eslen-Ziya H. The differential impact of COVID-19 on the work conditions of women and men academics during the lockdown. Gender Work Organ. (2021) 28:243–9. 10.1111/gwao.12529 32904915 PMC7461380

[25] Górska AM Kulicka K Staniszewska Z Dobija D. Deepening inequalities: what did COVID-19 reveal about the gendered nature of academic work? Gender Work Organ. (2021) 28:1546–61. 10.1111/gwao.12696 34219993 PMC8239576

[26] Amano-Patiño N Faraglia E Giannitsarou C Hasna Z. The Unequal Effects of Covid-19 On Economists' Research Productivity. Cambridge Working Papers in Economics [Internet] (2020). Available online at: https://www.dropbox.com/s/peljkeh0jkhpd0q/ProdGapEcon_2020AmanoFaragliaGiannitsarouHasna.pdf?dl=0 (accessed June 23, 2021).

[27] Nowakowska J Sobocińska J Lewicki M Lemańska Z Rzymski P. When science goes viral: the research response during three months of the COVID-19 outbreak. Biomed Pharmacother. (2020) 129:110451. 10.1016/j.biopha.2020.110451 32603887 PMC7309857

